# Cross-Sectional Study on the Prevalence of Intestinal Parasites and Associated Risk Factors in Teda Health Centre, Northwest Ethiopia

**DOI:** 10.5402/2013/757451

**Published:** 2013-08-12

**Authors:** Abraraw Abate, Biniam Kibret, Eylachew Bekalu, Sendeku Abera, Takele Teklu, Aregawi Yalew, Mengistu Endris, Ligabaw Worku, Zinaye Tekeste

**Affiliations:** School of Biomedical and Laboratory Sciences, College of Medicine and Health Sciences, University of Gondar, Gondar, Ethiopia

## Abstract

*Objective*. To assess the magnitude of intestinal parasitic infection and associated risk factors in Teda Health Centre, Northwest Ethiopia. *Method*. A cross-sectional study was conducted in Teda Health Centre from February to April, 2011. Stool samples were collected from 410 study participants and analysed by direct wet mount and formal ether concentration techniques. Furthermore, sociodemographic data were collected by using standardized questionnaire. *Result*. The overall prevalence of intestinal parasitic infection in this study was 62.3%. *Ascaris lumbricoides* was the most predominant parasite (23.2%) followed by *Giardia intestinalis* (12.4%), *Entamoeba histolytica/dispar* (4.6%), *Schistosoma mansoni* (8.9%), hookworm (6.6%), *Hymenolepis nana* (1.5%), *Enterobius vermicularis* (0.4%), and *Strongyloides stercoralis* (0.2%). Absence of toilet and hand washing after toilet was shown to be associated with intestinal parasitic infection (*P* < 0.05 for both). Furthermore, swimming and less shoe wearing habits showed a significant prevalence of *S. mansoni* and hookworm infections, respectively. *Conclusion*. The present study showed high prevalence of intestinal parasitic infection in the study area. Absence of toilet and hand washing after toilet was found to be associated with intestinal parasitic infection. Therefore, there is a need for integrated control programme to have a lasting impact on transmission of intestinal parasitic infection.

## 1. Background 

The intestinal parasites can be protozoan or helminth living within the body. Generally, these parasites are more common in tropics and subtropics than elsewhere in the world [1, 2]. It is closely associated with low income, poor personal hygiene, environmental sanitation, lack of pure water supply, limited access to clean water, tropical climate, and low altitude [1, 2].

*Entamoeba histolytica/dispar, Ascaris lumbricoides,* hookworm, and *Trichuris trichiura* are among the most common parasites in the world [2]. According to the World Health Organization (WHO) estimates, there are 800–1000 million *A. lumbricoides,* 700–900 million Hookworm infections, 500 millions *T. trichiura*, 200 million *Giardia intestinalis,* and 500 million *E. histolytica/dispar* cases globally [3]. Despite recent effort to control intestinal parasite infections, the diseases are still the leading causes of mortality and morbidity in the world [2].

In Ethiopia, intestinal parasitic infections are the major causes of mortality and morbidity causing a series of public health problems such as malnutrition, anaemia, and growth retardation as well as higher susceptibility to other infections [4–7]. Poor environmental sanitation, irrigation, overcrowding, resettlement, and low altitude were suggested to be responsible for high prevalence of intestinal parasitic infection in the country [2, 8–12]. However, prevalence of intestinal parasitic infections and the possible risk factors for intestinal parasitic infections were not illustrated in several localities of Ethiopia particularly in the study area. Therefore, the aim of this study was to assess the magnitude of intestinal parasitic infection and related risk factors in Teda Health Center. The research findings could provide data on the distribution and prevalence of intestinal parasites and assist in proposing strategies to protect those groups that might be at risk of intestinal parasitic infection.

## 2. Materials and Methods

### 2.1. Study Area and Population

The study was conducted from February to April 2011 in Teda Health Center which is found 24 km from Gondar town, Northwest Ethiopia. Teda town has an average altitude of 1800–2600 meters above sea levels, and the mean rainfall is 771–1160 mm. The town has two primary schools, one high school, one college, and one health centre. 

A total of 410 individuals participated in the study. Individuals, who had no history of anti-intestinal drug/s in the two weeks prior to screening, absence of any other serious chronic infection and those who had an ability to give stool samples were included in the study. 

### 2.2. Data Collection and Processing

Sociodemographic data were collected using standardized questioners. Stool specimens were collected using clean plastic sheet from all the study participants at Teda Health Center. Before starting the actual work, the specimens were checked for serial number, quantity, and procedures of collection. Furthermore, quality of reagents and instruments was checked by experienced laboratory technicians. Both saline wet mount and formal ether concentration techniques were used for examination of intestinal parasites as described by the WHO [13]. 

### 2.3. Data Analysis

The collected data were checked for completeness and analysed using Statistical Package for Social Sciences (SPSS) (16.0 version) and Simple Interactive Statistical Analysis (SISA) software. Chi square (*χ* ^2^) was used to determine association. Values were considered to be statistically significant when *P* values were less than 0.05.

### 2.4. Ethical Considerations

The study protocol was reviewed and approved by the Ethical Review Committee of Department of Medical Laboratory Sciences, University of Gondar. Written informed consent was obtained from all study participants and mothers/caretakers of children under 18 years old who participated in the study after explaining the purpose and objective of the study.

## 3. Results

### 3.1. General Characteristics of the Study Participants

A total of 410 subjects participated in this study, among them 198 (48.3%) were males and 212 (51.7%) were females. One hundred fifty one (36.8%) study participants were age group ≤14 years (Table 1). About 184 (44.9%), 143 (34.9%), 71 (17.3%), and 12 (2.9%) of the study participants were illiterate, elementary school students, high school students, and college or above, respectively (Table 1). Furthermore, 112 (27.5%), 129 (31.5%), 82 (20%), and 87 (21%) of the study participants get water from well, river, spring, and pipe water, respectively (Table 1). 

### 3.2. Prevalence of Intestinal Parasitic Infection

The overall prevalence of intestinal parasitic infection in Teda Health Center was 62.2% (255/410). Out of the 198 (48.3%) male and 212 (51.7%) female study participants, 62.1% (123/198) and 62.3% (132/212) were positive for at least one intestinal parasite, respectively. However, there was no significant association between sex and intestinal parasitic infection (*P* = 0.97) (Table 1).

About 65.8%, 61.1%, 57.7%, and 41.7% of the study participants categorized under illiterate, elementary school, high school, and college educational categories were positive for intestinal parasitic infection, respectively. However, there was no significant association between intestinal parasitic infection and education status (*P* = 0.28) (Table 1).

**Figure 1:**
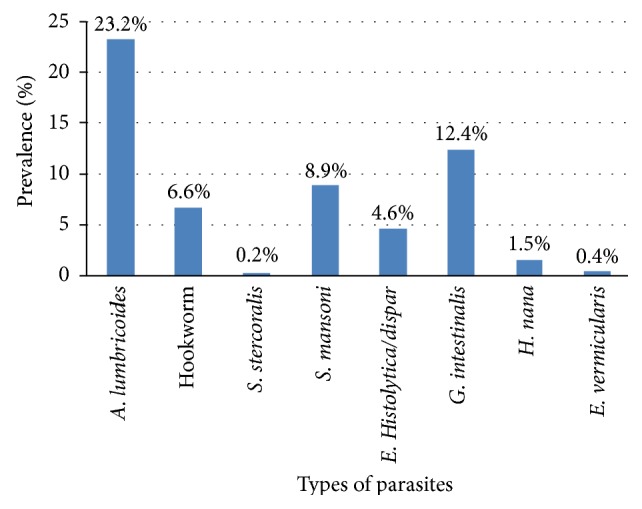
Prevalence of single parasitic infection in the study participants at Teda Health Centre from February to April, 2011.

**Figure 2:**
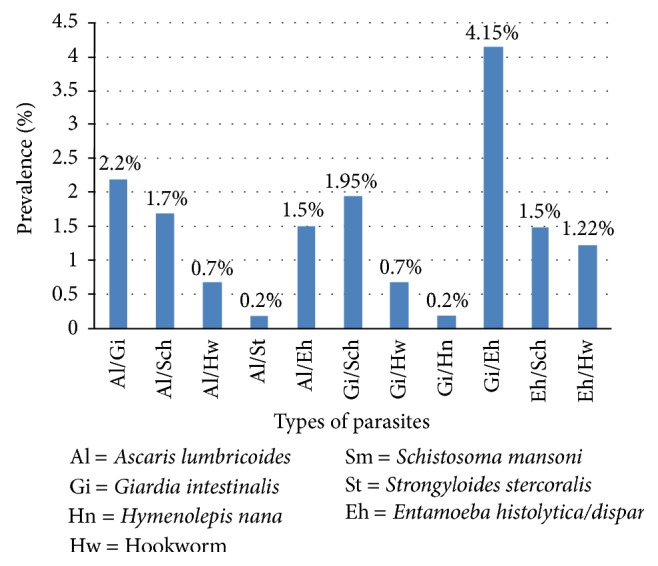
Prevalence of multiple parasitic infections in the study participants at Teda Health Centre from February to April, 2011.

Of the total study participants, about 4.6%, 12.4%, and 1.5% of the study participants were infected with *E. histolytica/dispar*, *G. intestinalis*, and *Hymenolepis nana*, respectively (Figure 1). *Ascaris lumbricoides* (23.2%) was the predominant parasite followed by *G. intestinalis* (12.4%) and *Schistosoma mansoni* (8.9%) (Figure 1). Furthermore, there were 2.20%, 1.70%, 1.95%, and 1.22% *A. lumbricoides* and *G. intestinalis*,* A. lumbricoides *and *S. mansoni*, *S. mansoni* and *G. intestinalis*, and hookworm and *E. histolytica/dispar* cases, respectively (Figure 2).

### 3.3. Associated Risk Factors

There was a higher percentage of intestinal parasitic infection in individuals who wash their hand before meal (64.2%) than those who do not (60%); the difference was not statistically significant (*P* = 0.076) (Table 1). Furthermore, there was a significant association between hand washing after toilet with intestinal parasitic infection (*P* < 0.05). Study participants who had swimming habit in rivers and less shoe wearing habit showed a statistically significant association with high prevalence of *S. mansoni* and Hook work, respectively (*P* < 0.05 for all) (Table 1).

## 4. Discussion

The demonstration that there was high prevalence of intestinal parasitic infection in this study was inconsistent with studies conducted in other regions of the country [14, 15]. For instance, a study conducted among school children in Jimma [15] town showed lower prevalence (47.1%) of intestinal parasitic infection than in the present study. Furthermore, lower prevalence (34.2%)of intestinal parasitic infection was reported from a study conducted in Northwest Ethiopia [14]. In contrast, higher prevalence of intestinal parasitic infection was reported from Azezo (72.9%) [16], Jimma (83%) [15], and Southeast of Lake Langano (83.8%) [17]. The contradictory report on the prevalence of intestinal parasitic infection could be due to variation in awareness regarding transmission and prevention of intestinal parasites between study participants in this study and previous studies. 

In Ethiopia, prevalence of ascariasis was reported to be 37%, which is higher than the prevalence of ascariasis in the present study [18]. Furthermore, studies conducted in Gondar and Southeast of Lake Langano also reported 5.9% and 6.2% prevalence of *Ascaris lumbricoides, *respectively [14, 17]. Furthermore, the demonstration that there was 7.30%, 0.50%, 10.50%, 12.90%, and 17.30% prevalence of Hook worm species,* S. stercoralis, S. mansoni, E. histolytica/dispar, G. intestinalis*,* and H. nana *was not in agreement with previous studies conducted in Ethiopia [14, 15]. For instance, as compared to the present study, lower prevalence of Hook worm and *Schistosoma mansoni* were reported among school children in Gondar [14]. In contrast, Mengistu [15] reported high prevalence of Hook worm and *S. mansoni* in urban communities of South-western Ethiopia.

The present study did not find a significant difference between intestinal parasitic infections and hand washing practice before meal (*P* > 0.05). This was inconsistent with studies conducted in Gondar and Babile [14, 19], where there was significant association between intestinal parasitic infections and hand washing practice. This difference might be due to the usage of contaminated water by the study participants for different purposes.

Although slightly higher prevalence rate was reported among illiterates, there was no significant association between education status and intestinal parasitic infections (Table 1). Similar finding was reported from a study conducted in Zarima town [20]. Higher level of education is usually associated with higher level of hygiene which might reduce the prevalence of parasitoses. This was not shown in our study; this might be due to the few number of study participants with college and above education (2.9%). Furthermore, a significant difference was observed in hand washing practice after toilet and absence of toilet with intestinal parasitic infection. The contrary association between hand washing practice after toilet and intestinal parasitic infection might be due the habit of using only water for washing in the area and inappropriate handling of readymade foods and drinks, without washing their hands using soap/ash. 

Swimming and less shoes wearing habits showed a statistically significant association with prevalent *S. mansoni* and Hook worm infections, respectively (Table 1). These results were consistent with the reports by Endris et al. at Azezo [16]. The prevalence of *S. mansoni* was higher in study participants who had swimming habit (18.9%) than who did not. This might indicate the presence of infested water body/ies in the study area. However, age, sex and source of water did not show statistically significant associations with intestinal parasitic infections.

In conclusion, the present study showed high prevalence of intestinal parasites in the study area. Furthermore, absence of toilet and hand washing after toilet was found to be associated with intestinal parasitic infection. Swimming and less shoes wearing habits showed a statistically significant association with prevalent *S. mansoni* and Hook worm infections, respectively. Therefore, there is a need for integrated control program including periodic deworming, construction of public toilets, creating awareness regarding the importance of washing hands after toilet and the impact of swimming in contaminated water to have a lasting impact on transmission.

## Figures and Tables

**Table 1:** The prevalence of intestinal parasitic infection with respect to sociodemographic characteristics of the study participants at Teda Health Centre from February to April, 2011.

Characters	Positive		Negative		Total		P values	χ^2^
	Number	%	Number	%	Number	%		
Sex								
Male	123	62.1	75	37.9	198	48.3	0.97	0.89
Female	132	62.3	80	37.7	212	51.7		
Age								
≤14	89	58.9	62	41.1	151	36.8	0.639	1.69
15–29	87	66.4	44	33.6	131	31.9		
30–44	41	62.1	25	37.9	66	16.1		
>45	38	61.3	24	38.7	62	15.2		
Education status								
Illiterate	121	65.8	63	34.2	184	44.9	0.28	3.770
Elementary school	88	61.5	55	38.5	143	34.9		
High school level	41	57.7	30	42.3	71	17.3		
College and above	5	41.7	7	58.3	12	2.9		
Hand washing habit								
Before meal								
Yes	138	64.2	77	35.8	215	52.4	0.383	0.076
No	117	60	78	40	195	47.6		
After toilet								
Yes	144	80.9	34	19.1	178	43.4	<0.01^*≠*^	46.8
No	111	47.8	121	52.2	232	56.6		
Source of water supply								
Pipe	53	60.9	34	39.1	87	21	0.087	0.69
River	78	60.5	51	39.5	129	31.5		
Spring	54	65.9	28	34,1	82	20		
Well	70	62.5	42	37.5	112	27.5		
Toilet								
Present	106	56.4	82	43.6	188	45.9	0.02^*≠*^	4.99
Absent	149	67.2	73	32.9	222	54.1		
Shoe wearing habit∗								
Yes	7	4.2	161	95.8	168	41	0.04^*≠*^	4.17
No	23	9.5	219	90.5	242	59		
Swimming habit∗∗								
Yes	42	18.9	180	81.1	222	54.1	<0.001^*≠*^	17.0
No	10	5.3	178	94.7	188	45.9		

Overall	255	62.2	155	37.8	410	100		

^*^Only for Hook worm, ^**^only for *Schistosoma mansoni,* ^≠^significant difference.
